# The Most Social of Maladies: Re-Thinking the History of Psychiatry From the Edges of Empire

**DOI:** 10.1007/s11013-021-09723-8

**Published:** 2021-06-24

**Authors:** Claire Edington

**Affiliations:** 1 Associate Professor of History, University of California, San Diego, USA; 2 Department of History, Humanities and Social Sciences Building, 0104, 9500 Gilman Drive, La Jolla, CA 92093-1014 USA

## Abstract

This paper argues that the colonial experience was never just “out there” but was a constitutive feature of the global development of psychiatry and, indeed, of social medicine itself. I show how regional knowledge about psychiatry, produced in scientific exchanges across colonial Southeast Asia over four decades and culminating with the 1937 Bandung Conference, became part of new international approaches to health care in rural areas, and later, in developing nations. In particular, I discuss how the embrace of the agricultural colony as a solution to the problem of asylum overcrowding occurred at the same moment that colonial public health experts and officials were moving away from expensive, technocratic fixes to address indigenous health needs. Yet in the search for alternatives to institutionalized care, including forms of family and community support, colonial psychiatrists were increasingly drawn into unpredictable and unwieldy networks of care and economy. Drawing on research from Vietnam, this paper decenters the asylum so as to recast the history of colonial and postcolonial psychiatry as integral to the history of social medicine and global health. The paper then returns to Bandung in 1955, the site of another famous meeting in the history of Third World solidarity, to consider how the embrace of the “Bandung spirit” may provide new avenues for decolonizing the history of colonial and postcolonial psychiatry.

## Introduction

This article argues that the colonial experience was never just “out there” but was a constitutive feature of the global development of psychiatry and, indeed, of social medicine itself. Regional knowledge about psychiatry, produced in scientific exchanges across colonial Southeast Asia over four decades and culminating with the 1937 Bandung Conference, became part of new international approaches to health care in rural areas, and later, in developing nations. The 1937 Bandung Conference, especially, signaled the ways in which concerns of the international health community, like rural hygiene and primary care, came to influence regional-level health agendas and priorities, including those concerning the mentally ill. The embrace of the agricultural colony as a solution to the problem of asylum overcrowding, for example, occurred at the same moment that colonial public health experts and officials in the region were moving away from expensive, technocratic fixes to address indigenous health needs. Psychiatry as social medicine—at least as it was imagined in colonial Southeast Asia—promised to cut costs by relying on families to provide care and by disciplining a labor force adapted to the needs of the regional plantation economy.

The ruralization of psychiatry—framed as a return to the community and the rhythms of agrarian life—serves as an apt invitation for historians to cast their gaze to the social world of the colonial asylum. The role of caregiving outside formal psychiatric settings across the globe, for instance, has long been recognized by anthropologists. Yet this vital feature of psychiatric knowledge-making and practice remains largely obscured in histories of colonial psychiatry which tend to emphasize the hegemony of the colonial asylum as well as its frustrated limits. In contrast, my own research takes conversations happening outside the official orbit of institutions seriously as an essential aspect of psychiatric power in French colonial Vietnam. In *Beyond the Asylum: Mental Illness in French Colonial Vietnam* (Cornell University Press 2019), I examine the way medical experts, state institutions, and popular beliefs shaped the lives of the mentally ill throughout the late nineteenth and early twentieth centuries. I am centrally interested in how ideas about deviance and disease travel across cultures and what everyday exchanges between lay people and experts can tell us about the social history of Vietnam. Drawing on hundreds of patient case files from two asylums built by the French—the first, Biên Hoà, outside Saigon in 1919, the second, Vôi, outside Hanoi in 1934—I follow the movements of patients in and out of asylums and between prisons, poor houses, youth reformatories, hospitals, and family homes. Together, these individual patient itineraries challenge our notion of the colonial asylum as a closed setting where patients rarely left, run by experts who enjoyed broad and unquestioned authority. Instead they reveal just how porous the boundaries between the asylum and community could be. It is this movement between the worlds inside and outside the colonial asylum—indeed the blurring of boundaries between these worlds and the tensions that result—that form the central focus of my book.

Studying the social aspects of psychiatry from the edges of empire is not an attempt to foreground the periphery, the colony, as an act of recuperation. Rather it is an attempt to decenter the colonial asylum so as to cut—to slice through distinctions between Western and non-Western, traditional and modern, local and global, and to unbind these categories from the outset. Vietnamese understandings of madness were themselves an assemblage of indigenous and imported knowledge systems, and French attempts to establish psychiatry as an independent branch of scientific medicine, at home and abroad, were continuously undermined and delayed. The messiness extends further once we consider the asylum as one node in a larger network of disciplinary practices and institutions that tied the structures of Vietnamese private and public life together with those of the colonial state. Colonial expertise was not only French and not only institutionally sanctioned, it was heterogeneous and produced in exchanges between experts and laypeople. The arrival of asylums may have marked the beginning of psychiatry as a state-sponsored project in Vietnam, but it was inserted into a pre-existing field of knowledge and practice that it never fully succeeded in displacing.

Colonial Vietnam itself emerged as a node in growing regional health networks in the early decades of the twentieth century. These inter-imperial and, increasingly international, exchanges went far toward establishing Southeast Asia in the minds of indigenous and colonial experts alike as a coherent entity, a shared object of knowledge that could be investigated, managed, and exploited using similar methods. At the 1937 Bandung meeting, Southeast Asia figured centrally in international conversations about rural hygiene as a way to mitigate the effects of agrarian depression worldwide. These conversations about the indigenization of health care, particularly in the countryside, refracted back to Indochina, shaping the colonial public health landscape in important ways. By moving the mentally ill out of doors and back into the community, psychiatric experts made themselves dependent on patient families who did not always share the same appraisals of risk, illness, or responsibility. Instead, families pursued their own strategies that reflected a mixture of both homegrown and imported understandings about the cause of mental illness and its treatment, concerns over meeting social obligations and managing household precarity. The last part of the paper returns to Bandung in 1955, the site of another famous meeting in the history of decolonization and Third World solidarity. It speculates on what a decolonial reading of the 1937 meeting might look like and what such a perspective would contribute to our understanding of histories of social medicine and global health more generally.

## The View from Bandung

Bandung’s location near the volcano Tangkuban Perahu, named for a fishing boat (prau) turned upside down (Personal correspondance, Micale and Pols 2021), seems like the perfect vantage for taking a wide view of the field.

The 1937 Bandung Conference on Rural Hygiene, a meeting that historians now view as a milestone in the development of international initiatives focused on social medicine and rural public health. Organized by the League of Nations Health Organization (LNHO), this regional conference in West Java capped what historians Theodore Brown and Elizabeth Fee describe as a “surge of interwar interest in rural hygiene [that] in several ways foreshadowed the WHO’s famous Alma Ata Conference” (Brown and Fee 2008). A Conference on Rural Hygiene held in Geneva in 1931—organized in response to the poor living conditions of Europe’s predominantly rural population in the wake of World War I and the Great Depression—became the blueprint for the meeting in Asia, first proposed by Indian and Chinese delegations to the LNHO in 1932. In particular, the 1937 conference addressed concerns over what to do about the vast majority of Asia’s population that lived in poverty in the countryside, far from modern hospitals and with limited access to “modern” medicine. The conference stressed the importance of prevention—including public health education and the establishment of small clinics focused on maternal and child health—instead of expensive, technology-intensive curative approaches that remained outside the reach of most indigenous peoples. It also emphasized the need to pay attention to the languages, cultures, and traditions of local populations, as well as broader issues surrounding economic development and land reform.

As part of the conference, the French delegation from Indochina made a satellite visit to Lenteng Agung, where in 1933 two hundred calm, chronic psychiatric patients had been transferred from the asylum at Buitenzorg, and put to work in the fields under minimal supervision. Following the famous Belgian model at Gheel, seventy patients were later placed in the homes of villagers who housed and fed patients in exchange for their labor. Not only did this hybrid model of care encourage patients to readapt to normal Javanese village life, but it also helped to relieve the tremendous financial burden of caring for the insane who no longer seemed to benefit from confinement. The initiative harmonized with the overall mood of the conference; it emphasized the treatment of patients in familiar (i.e., rural) settings, signaling a shift away from the deployment of technical expertise in institutions and toward a more social, integrated perspective on health and hygiene. It was psychiatry as social medicine in action and explicitly touted as a model for others to follow.

In fact, the French had long been interested in Dutch experiments with the use of therapeutic labor for patients (Pols 2012; Schrauwers 2001). Following a number of study trips to neighboring Java, they chose to follow the Dutch lead by also organizing asylums in Indochina as large agricultural colonies where patients could work the land on the path to healing and eventual liberation. The model of the agricultural colony promoted a view of psychiatric rehabilitation that blurred the distinctions between patients and laborers, between spaces of confinement and release. In simulating real life outside the walls of the asylum, it was designed to create a kind of continuity between the discipline of institutional order and social life in the community, a kind of “social re-education” (Ernst 2016). With the expansion of plantation economies in the region, institutions that transformed troublesome “natives” into productive colonial subjects appeared unusually promising to both Dutch and French colonial administrations (Edington and Pols 2016).

This movement of psychiatric knowledge and practice across colonial empires increasingly took place within the framework provided by international health organizations, which created new opportunities for site visits, scientific journeys, and medical exchanges across Southeast Asia. The Far Eastern Association of Tropical Medicine (FEATM)—founded in 1908 by American medical experts in the Philippines—became an important forum for information sharing and inter-imperial cooperation. In addition to establishing region-wide quarantine policies and an anti-beriberi campaign, meetings of the FEATM also addressed the management of the mentally ill including the elaboration of “tropical psychoses” such as amok and latah. The League of Nations Health Organization and International Labor Organization, as well as the Rockefeller Foundation, also projected their considerable influence throughout Southeast Asia during the interwar period. (Tomoko 2015) By the early 1930s, in the midst of the global economic depression, Southeast Asia became increasingly central to debates about what to do about the human and economic fall-out of rural poverty. Experiments in rural hygiene across Asia—which mobilized new scientific knowledge and advances in sanitary engineering to promote gradualist, indigenous development—seemed to hold the key to solving similar subsistence crises elsewhere, including Eastern and Central Europe. The Bandung meeting, to borrow Sunil Amrith’s words, signified a “leap in scale when it came to thinking about problems of health – especially rural health within Asia” (Amrith 2014, 170). Southeast Asia had ascended to the global stage.

Lenteng Agung was one such experiment in rural hygiene. While the French ultimately chose not to adapt this model for use in Indochina, it nevertheless represents several key transformations taking place at Bandung that grafted imperial and international visions for health onto each other. First, it represented a transformation of understandings about the “rural.” For visitors to Lenteng Agung, the enthusiasm for stand-alone agricultural colonies stemmed in part from older nineteenth century visions that romanticized the countryside as a site for the restoration of authority, order, and social discipline in the wake of the 1848 revolutions throughout Europe (Ceri 1991). Indeed this vision had supported the earlier proliferation of agricultural colonies throughout the region not only for psychiatric patients, but also for political prisoners, juvenile delinquents and lepers (Monnais 2008, Bergen 2018). At the Bandung meeting, however, international experts articulated new visons of the “rural” that saw the revitalization of the countryside as crucial for the well-being of the nation. This view challenged the perspective adopted by colonial regimes which saw the extension of Westernized health care to rural populations as costly and, ultimately, ineffective. Instead it figured rural areas as sites which required active intervention, emphasizing the need to pay attention to local “culture,” however, narrowly defined. As experts, they saw their role as one of providing education to rural inhabitants on how to transform their own living environment, from improved diets to healthier homes. Some even hoped that rural reconstruction would result not only in economic uplift but would also help to facilitate the growth of national consciousness (Naono 2014).

Second, Lenteng Agung took the form of a “small-scale demonstration project,” a key aspect of international health in Asia that sought to apply scientific principles to the transformation of rural society. The appeal of these demonstration projects, like the modern pilot-study, allowed for flexibility and improvisation, tailored to local conditions. With respect to the mentally ill, decentralization would prove key even if the supervision of the expert would remain paramount. As the final meeting report from Bandung emphasized, “Although the care of the insane should be decentralized, it is essential to have a central post from which an expert psychiatrist may exercise control over admission stations, asylums, colonies and the boarding-out of patients…Propaganda and education must be applied to combat the idea, widespread in Eastern countries, that mental disease are different from other diseases” (Report of the Intergovernmental Conference of Far-Eastern Countries on Rural Hygiene, held at Bandoeng, Java, August 3rd to 13th, 1937, 115). These demonstration projects were also decidedly modest in their ambitions, promising incremental change not likely to disrupt the political status quo. Finally, as social experiments, they were limited in scale and therefore cheap (Amrith 2014). Lenteng Agung, for instance, freed up vital space at the asylum for more acute cases by transitioning patients to “households” in a model village overseen by a health official.

The impetus to get patients out of asylums and into transitional care settings was appealing at a moment when colonial governments faced the imperious need to cut costs. The same mechanisms putting survival pressure on individual households also squeezed colonial states operating on a shoe-string budget. Indeed, in Indochina, the financial situation was dire and asylums were expected to be self-supporting. By 1934, for instance, nearly a third of all patients at the Biên Hòa asylum were put to work. As much as asylum directors emphasized that patient labor was voluntary and therapeutically effective, their labor was nevertheless vital for keeping these institutions afloat. And yet, by drawing the practice of psychiatry out of the institution and into the social world, colonial experts found themselves having to continually negotiate the care of patients and defend their professional acuity and judgment. In this second half of the paper, I show how “psychiatry as social medicine” as it was practiced on the ground requires a concomitant shift in the writing of the history of colonial psychiatry, to reorient the center of the story from the institution itself to those exchanges that criss-crossed its walls.

## The View from Colonial Vietnam

Colonial asylums could not and did not operate in isolation. Their reliance on the public to not only present patients for treatment but also to take care of patients upon their return home meant they were continually forced to negotiate the terms of patient entry and release. At every turn, asylum administrators in interwar Indochina were beset with the challenges of failed surveillance and interpersonal violence produced by patient overcrowding, diminishing budgets, and problems with staff recruitment and retention, which only deepened their dependency on extra-institutional forms of support. Vietnamese families, meanwhile, pursued their own strategies in ways that both facilitated and constrained the ambitions of colonial psychiatrists. In some instances, families eagerly solicited the services of state-run institutions and in others, attempted to shield family members as much as possible from the outside world.

Rather than abandon their relatives to the care of French experts, families would often write to doctors, visit patients, and demand their release or transfer to other institutions. In letters to asylum directors, family members offered their own opinions about the patient’s condition and at times asserted that their loved ones had, in fact, improved enough to come home. These family assessments did not always square with those of experts and, at times, would generate significant conflicts. Debates revolved around the mental health of the patients but also the capacity of the families to assume their care upon release and the asylum itself as the most appropriate site for treatment and rehabilitation. Vietnamese families, armed with a different set of cultural beliefs and healing traditions, nevertheless engaged with French psychiatry to find common space for talking about mental illness.

Yet even when French doctors and Vietnamese families broadly agreed about the presence of mental illness that required treatment, important differences emerged when it came to interpreting the origins of the affliction and what should be done about it. The cultural content of mental illness itself—how symptoms of psychiatric distress were experienced and expressed by patients and how they were interpreted by those closest to them—also posed serious challenges for colonial doctors who insisted on diagnosing disorders based on classification schemes developed in France. While the categories of normal and abnormal continued to be framed in Western terms, the content of these categories critically relied on the kinds of information Vietnamese were willing or thought relevant to tell French doctors. In this way, psychiatrists did not come to Indochina finding a blank slate, devoid of any local expertise or ways of coping with madness. Rather, they encountered forms of care in the community that long predated French occupation and that would continue to play an important role in the expansion of the formal asylum system.

The stories of patients and their families tend to reinforce Michael MacDonald’s observation in *Mystical Bedlam*, his magisterial work on the history of madness and healing in seventeenth-century England, when he writes, “Madness is the most solitary of afflictions to the people who experience it; but it is the most social of maladies to those who observe its effects” (Macdonald 1981, 1). In order to draw out these ‘social’ qualities of madness, ones that are often obscured or lost in colonial histories, patient case files must be read for what they can tell us about life outside the asylum as much as the life inside its walls. Indeed these files have the potential to uncover some of the texture of everyday life under colonialism: the practices and expectations for caregiving in the community, the strategies of dependence and survival that guided the decision-making of households, and the variety of social roles performed by patients which shaped their experiences on either side of psychiatric institutions: as farmers and dock workers, prostitutes and petty thieves, parents and children.

Throughout the early twentieth century, Vietnamese families sought the care of both local *and* foreign experts but they also discovered recipes for home remedies in the pages of popular scientific journals that appeared in the 1920s and 30s as part of Indochina’s vibrant press culture. This public sphere played host to a new marketplace for ideas about mental illness—from melodramas about suicide and broken hearts to advice columns which allowed concepts that in scientific language might be too difficult and abstract for the common reader to be recast in the form of confessions and expert response. Whereas colonial French sources tended to dismiss traditional Vietnamese medicine as quack or non-scientific, these Vietnamese-language journals provided a space where Western-trained Vietnamese doctors could communicate with each other and the public (Monnais and Tousignant 2006). Rather than cast aside indigenous explanatory models of illness, these authors explicitly drew on local conceptions of health and disease in order to reach the average reader. Vietnamese understandings of mental illness bear the influence of traditional Chinese medicine, particularly in terms of the blurring of psychological and physical health: emotional states are closely tied to physical disturbances and vice versa. For some Vietnamese writers, this environmental framework was not necessarily incompatible with Western (and especially Hippocratic) ideas about disease, and instead insisted on points of conceptual overlap.

This is perhaps seen most clearly in glossary entries, which appeared in popular scientific journals. Here Vietnamese experts attempted to square Sino-Vietnamese medical knowledge—itself a prior assemblage of Confucian, Taoist, Buddhist, and folk traditions—with French diagnostic categories rooted in an entirely different nosology based on distinctions between mind/body. In the typology of mental illness that emerged, what is perhaps most striking is the use of Vietnamese idioms of “weakening nerves” and “poisoned blood” to describe various mental disorders while at the same time offering an equivalent French clinical designation (appearing in parentheses in the text, in italic font). Even as doctors made use of colloquial expressions and everyday experiences, they nevertheless sought to draw out in a more scientific fashion the distinctions between specific syndromes, often by making reference to more advanced French medical terminology. These French terms would act as temporary placeholders until satisfactory Vietnamese equivalents emerged alongside and would eventually assume their place in the scientific literature (Monnais 2012, Trong 2009).

These sources help make sense of family decisions around treatment contained within the pages of patient case files. What they learned from the popular press may have shaped their expectations of what asylum care may look like and whether it was even necessary. It may also have reinforced a sense of how the practice of vigilant self-discipline, and even the performance of caregiving itself, came to be considered a vital part of what it meant to be modern and Vietnamese. The failure to avoid mental illness usually implied a bad family history, weak nerves, or an inability to cope with the stresses of modern life. These sources also give us a glimpse into a society in flux—indeed what is perhaps most striking is how mental illness was mobilized to register critiques of colonial society: the avarice of Vietnamese elites, the erosion of traditional gender norms, the superstitious practices of rural villagers, the romantic idealism of young women, and the plight of the urban poor wrought by the destructive forces of French colonization.

Across these examples we see how knowledge about mental illness escaped the official channels of medical training and institutions in the colony. Alternative forms of expertise were staked on common sense and lived experience—tethered to local cosmologies and cosmopolitan attitudes about self-regulation and social responsibility. Rather than an exercise in the straightforward translation, or even appropriation, of medical concepts across cultures, these exchanges became the site of a new assemblage of knowledge about mental illness, a composite of different epistemologies. By playing host to a public sphere that brought new ideas to new audiences, for instance, the Vietnamese press allowed diverse understandings about the provenance and progression of mental illness to be assembled in such a way that was meaningful to its reading public. In so doing, it played a vital role in not only the circulation of knowledge but also in making a new kind of localized knowledge possible.

Reading patient case files alongside works from the Vietnamese popular press allows us to see just how far ideas about psychiatry and mental illness traveled outside the colonial asylum, and the extent to which they resisted the racist impulses of colonial medicine. Postcolonial scholars often refer to these spaces of negotiation as “contact zones” and the forms of knowledge they produced as by turns hybrid, contingent, unstable, and situated. The point is to decolonize narratives of scientific progress and Western modernity, to take seriously subaltern forms of knowledge, and to lay bare the power relationships which make labels like “modern” or “abnormal” seem natural and inevitable in the first instance (Anderson 2014, Chiang 2015, Hashimoto 2013, Pols 2007, Wu 2016).

## Back to Bandung

As the historian Randall Packard reminds us, while the 1937 Bandung conference may have represented the culmination of the interest in rural reconstruction and hygiene outside Europe, it also reflected the continued entanglement of international health organizations and colonial medicine. Despite the attention to the social and economic origins of poor health in the region, he writes, “At almost every turn, conference participants backed away from recommendations that would have radically changed the status quo in order to improve rural hygiene in their countries” (Packard 2016, 85). The failure of colonial governments to embrace more ambitious plans for development frustrated indigenous leaders who envisioned more sweeping, and revolutionary, reforms. Moreover, the dissolution of the League of Nations in advance of the Second World War, alongside the technological revolution which heralded the arrival of DDT, spelled the death knell for this more romanticized vision of rural health as the guiding ideology of international health. Instead, the World Health Organization, founded in 1946, came to focus on vertically organized, resource-intensive eradication campaigns (Amrith 2006; Stepan 2011). In the absence of magic bullet therapies for the treatment of most mental disorders (chlorpromazine for schizophrenic patients would not be discovered until 1952), social medicine, with its emphasis on prevention and community care, retained a primary role.

In histories of social medicine and global health, the 1937 Bandung conference has undergone a recent resurgence of scholarly interest. It is viewed as an important precursor to the later 1978 Alma Ata conference where the World Health Organization articulated primary health care as the goal of international health (Guénel 2012, Litsios 2014; Brimnes 2019).

Yet what remains strikingly absent from the narration of this history is the mention of another major conference held at Bandung, eighteen years later in April of 1955. Delegates to this meeting included state leaders, intellectuals, and activists of color from twenty-nine states across Asia, Africa, and the Middle East. Together, they represented 1.5 billion people or two-thirds of the world’s population. It was a diverse mixture of interests: some delegates represented colonized regions, others newly independent countries like Indonesia, and still others were embarking on their own colonial projects. Some were communist, others pro-West, and yet they all shared a common history of Western imperialism and oppression. In his opening address to the conference, Sukarno emphasized his country’s motto “Unity in Diversity” (Bhinneka Tunggal Ika) remarking, “Sisters and Brothers, Indonesia is Asia-Africa in small.”

The 1955 meeting at Bandung was a landmark occasion for the expression of “Third World” solidarity, referred to subsequently as the “Bandung Spirit.” It rejected both past and future imperialisms, condemning the ascendant power of the United States and Soviet Union. Instead it posited a different vision of a world order, a more virtuous and positive one, committed to human rights and self-determination, economic cooperation and cultural exchange, and world peace (Lee 2019). It explored the possibilities of Afro-Asianism—or what Frantz Fanon referred to as the “Bandung-Accra Axis”—as a potent new world-historical force. As Fanon wrote in 1958, “The awakening of the Asian and African masses is not idle talk. Its effects translate into concrete achievements that colonialists no longer have it in their power to ignore. The movement that set out from Bandung in April 1955 knows no stopping. Its all-powerful waves are sweeping away, one after the other, the most deeply rooted imperialist strongholds” (Fanon 2018, 628).

For scholars reflecting on the legacies of the 1955 Bandung meeting, its failures have proved less generative than using the event as an opportunity to sketch out a decolonial framework or what Christopher Lee describes as “Bandung as Method.” For Lee, the meeting represents an invitation: (1) to think about the Global South as an imaginative political geography, one that does not take the West as the essential reference point and instead explores South–South connections that may bypass the West altogether; (2) to move beyond nation-state narratives and instead emphasize interconnections across continents as well as historical periods; and (3) to figure Bandung as not just an event but also a site of becoming where indigenous actors articulated different aspirational futures and inserted a new form of historical agency onto the world stage (Lee 2019).

What happens when we interpret the 1937 Bandung meeting through a postcolonial lens? This paper represents an attempt to use Lee’s “Bandung as Method”—to decenter Western histories of psychiatry and social medicine by foregrounding the periphery, in this case Southeast Asia. The regional development of psychiatry bore the marks of both inter-imperial and international exchanges that experimented with the principles of rural (in this case, mental) hygiene and community-based care. At Bandung in 1937, these local practices and expertise were transformed into a new international discourse on health. Furthermore, decentering the asylum as *the* classic instantiation of Western colonial power shows just how unstable and fragmented psychiatric power was in reality, exposing the range of indigenous participation in shaping the production of psychiatric knowledge and practices in Indochina. This perspective requires historians of colonial psychiatry to read more traditional archives, i.e., patient case files, in new ways, for what they can tell us about life outside the asylum, as well as expanding what counts as the “archive” in the first place. Looking at the Vietnamese popular press, for instance, provides vital clues as to what patient families understood to be the possible origins and treatment of mental disorders well as how the language of mental illness was mobilized to talk about the promises and perils of modernity and the exploration of alternative futures for a decolonized Vietnam.

Furthermore, thinking about interconnections across time—or what Lee describes as “layered histories”—draws important attention to the legacies of colonial mental health practices and institutions which can be rendered opaque in the origin stories of global mental health. Many of these colonial asylums outlived the empires that built them. They were the training grounds for native doctors who would take up the reins after the wars of decolonization, as well as French doctors, like Pierre-Marie Dorolle, who would abandon their posts to assume leadership positions at the World Health Organization after World War II (Wu 2015). While the history of Western postwar psychiatry is often described as undergoing a profound paradigm shift during this period, it also clearly reflects continuities with the agenda outlined at Bandung. After the war, psychiatry sought to shed its colonial racist past by transforming from an instrument of imperial subjugation into a forward-looking science dedicated to expanding treatment and care for those in need. The concept of “world citizenship,” as articulated by the first director-general of the WHO Brock Chisholm, provided the framework in which this new democratizing project of social psychiatry was imagined. It took as its starting point a universalist notion of mental disorders, one that, while acknowledging variation across countries and cultures, nevertheless insisted on the possibility of applying common diagnostic criteria, thereby paving the way for a more inclusive notion of mental pathology. Much like the 1937 meeting, this new paradigm emphasized the importance of intersectoral, international collaboration as the basis for expanding epidemiological research on both local and global scales. Harry Wu nevertheless describes this project as an “idealistic effort,” one which “sought to conform to the central dogma of the WHO, which envisioned health as a basic human right of all ‘world citizens’ before this ideal was absorbed into global developmentalism” (Wu 2016). And yet, while mental health was soon marginalized as a priority within the WHO, efforts by psychiatric and development experts alike to incorporate mental health as a component of primary care gained traction well before Alma Ata. Even if the emphasis on state-based mental care remained fixed on the institution, examples from Vietnam and other settings across Asia point to the continued reliance on forms of family and community care throughout the era of decolonization and postcolonial nation-building, including the use of indigenous pharmacopeia and reliance on ritual healers. Moreover, the neutralization of the more revolutionary potential of social medicine through discourses of “development” and “world citizenship” represents yet another continuity with the pre-war period.

Hardly the “world-making” revolution was envisioned by Fanon. However, this period also saw the roots of a more radical project, one which sought to decolonize the discipline through the development of a new transcultural psychiatry. Perhaps the most famous example, the Fann psychiatric clinic established in 1956 in Dakar, sought to integrate Western-based psychiatric care with local social traditions and healing rituals, inviting traditional healers into the space of the clinic. As Katie Kilroy-Marac has recently noted, this project was plagued with internal tensions over whether an emphasis on cultural differences laid the groundwork for a truly liberatory project, breaking free of Western colonial prejudice inherent in psychiatric universalism, or whether it merely served to further entrench notions of cultural essentialism (Kilroy-Marac 2019).

Attention to these layered histories of colonial and postcolonial histories of psychiatry, and especially their role in the development of social medicine, helps to disentangle the genealogies of global mental health as previously urged by Anne Lovell in the pages of this journal (Lovell 2019). But I am also suggesting that we include the 1955 Bandung Conference as an essential plot point in the unraveling of this story. Fanon’s formulation of a “Bandung-Accra” axis for Third Worldism, for instance, should be taken as an invitation to take more seriously the project of connecting histories of psychiatry across the Global South, ones that are not necessarily routed through Geneva, or even institutionalized in the form of Dakar’s Fann clinic or Nigeria’s Aro village (Heaton 2013). Instead, what might happen if we identify conceptual and interpretative frameworks that help us to address “local understandings of suffering and local cultural repertoires to overcome them” (Micale and Pols 2021) on their own terms, rather than as variations on a universal theme? What if we attend to how these experiences of suffering are rendered legible and legitimate both inside *and* outside the clinic? What if we pursued global connections not in the (frustrated) universal deployment of psychiatric categories, or their capture in large-scale epidemiological studies, but in terms of shared experiences of trauma, dislocation, and survival?

One powerful example is provided in a new volume on *Trauma in Asia*, edited by Mark Micale and Hans Pols, which insists on the necessity of taking Asian experiences of trauma “on their own terms” and “to work toward the construction of a model of psychological trauma that is truly globalized—globalized, not just factually and geographically, but also conceptually and interpretatively” (Micale and Pols 2021, X). In particular, the volume shifts emphasis away from severe mental illness treated in hospital settings and toward an examination of mental disorders that are collectively experienced in response to traumatic events such as natural disasters, war, famine, state violence, and the creative responses mounted by communities through shared rituals of healing and rebuilding.

In this same volume, Narquis Barak examines the divergent histories of caring for traumatized civilians and soldiers in the United States and Vietnam. Whereas Post-Traumatic Stress Disorder (PTSD) was developed in the US to describe the trauma of American soldiers returning from the Vietnam War, PTSD as a diagnostic category remains virtually absent in Vietnam. Instead, Vietnamese views of trauma reflect the “layered histories” of Chinese, French, and Soviet influence in the region. Furthermore, by giving separate names for patient symptoms attached to the specific type of American ordinance or chemicals responsible for the initial traumatic episode, the effect is to render visible the specific forms of violence enacted on patients, rather than attribute their suffering as an individual failure to adapt to their social environment. Research on the trauma experienced by Vietnamese refugees, and their children, provides yet another avenue for pursuing connections across both time and space that may trouble or elide the dominance of Western psychiatric expertise altogether (Silove 2007, Hinton 2003).

## Conclusion

The practice of Vietnamese psychiatry today reflects the myriad influences which have shaped ideas about the origins of mental distress and the expression of symptoms, local understandings of the self and expectations for caregiving, whether from families or the state. Even though the enthusiasm for social medicine at the 1937 Bandung meeting faded quickly from view on an international level, its reverberations can be seen in the constitution of “health brigades” by several Asian countries in the 1950s, as well as the enduring embrace of traditional medicine within national health care systems, including Vietnam, as a mark of postcolonial nationalism. In a curious twist, while the outpatient model for recovered psychiatric patients at Lenteng Agung (mentioned above) ultimately failed to gain traction under the French, it was eventually adopted by the government of South Vietnam in 1964 when the Mental Health Bureau of the Vietnamese Ministry of Health created a rehabilitation village on the periphery of the grounds of the former Biên Hòa asylum. It placed seventy patients set to leave the hospital after three or four years of treatment in a kind of halfway institution, where they were left to earn a small living by hiring themselves out to farmers in the surrounding area. Just as with the Dutch experimental model at Lenteng Agung, the objective was to help patients prepare for reintegration into normal life by equipping them with important skills so that they would no longer be a burden on their families or an object of pity in their communities. The hospital staff deemed the project so successful that the capacity of the village was eventually doubled in 1970.

The Biên Hòa asylum is still the site of a functioning psychiatric hospital in Vietnam today, in fact, the nation’s largest and 2019 marked its centennial. It forms part of a network of regional psychiatric hospitals and, increasingly, community-based mental health care centers, the result of a massive influx of development money into Vietnam from the global public health community keen to set local policy agendas. Today, as in the colonial past, scientific expertise continues to be negotiated in the clinic and in policy circles and social stigma remains a major barrier to care. At the same time, it is widely observed that families will often stay with patients at the psychiatric hospital throughout the duration of their treatment, playing the vital role of caretakers even within the walls of the institution (Tran 2012, 2017).

Only by considering the history of psychiatry as also a history of social relationships do we get a true sense of the reach of psychiatric norms into daily life, and the range of actors who participate in deciding the fate of the mentally ill. To the extent these exchanges draw on different epistemologies and ethics of caregiving, reflecting regional health agendas *and* global circulations of expertise, they test not only assumptions about the universality of psychiatric knowledge but also notions of where we understand that knowledge to be created in the first place. As a historical method, psychiatry as social medicine entails expanding the archive to capture the full social context in which determinations of mental distress are formulated, negotiated, and resisted. It also requires looking beyond received epistemological categories—especially those developed in the West—in order to find new bases for comparison and connection that acknowledge the diversity of human suffering, the importance of caregiving, and the ways the past infuses the present, materializing in ghostly presences and intergenerational memories. Much like Frantz Fanon writing of the promise of the 1955 Bandung Pact, which marked the “advent of peoples, unknown only yesterday, onto the stage of history,” a truly decolonial history of psychiatry promises a vital step toward this “world process of humanization” (Fanon 1967, 146).
